# Detection of mental imagery and attempted movements in patients with disorders of consciousness using EEG

**DOI:** 10.3389/fnhum.2014.01009

**Published:** 2014-12-12

**Authors:** Petar Horki, Günther Bauernfeind, Daniela S. Klobassa, Christoph Pokorny, Gerald Pichler, Walter Schippinger, Gernot R. Müller-Putz

**Affiliations:** ^1^Laboratory for Brain-Computer Interfaces, Institute for Knowledge Discovery, Graz University of TechnologyGraz, Austria; ^2^Department of General Pediatrics, Medical University of GrazGraz, Austria; ^3^Institute for Theoretical Computer Science, Graz University of TechnologyGraz, Austria; ^4^Albert Schweitzer ClinicGraz, Austria

**Keywords:** EEG, mental imagery, attempted movements, passive movements, disorders of consciousness

## Abstract

Further development of an EEG based communication device for patients with disorders of consciousness (DoC) could benefit from addressing the following gaps in knowledge—first, an evaluation of different types of motor imagery; second, an evaluation of passive feet movement as a mean of an initial classifier setup; and third, rapid delivery of biased feedback. To that end we investigated whether complex and/or familiar mental imagery, passive, and attempted feet movement can be reliably detected in patients with DoC using EEG recordings, aiming to provide them with a means of communication. Six patients in a minimally conscious state (MCS) took part in this study. The patients were verbally instructed to perform different mental imagery tasks (sport, navigation), as well as attempted feet movements, to induce distinctive event-related (de)synchronization (ERD/S) patterns in the EEG. Offline classification accuracies above chance level were reached in all three tasks (i.e., attempted feet, sport, and navigation), with motor tasks yielding significant (*p* < 0.05) results more often than navigation (sport: 10 out of 18 sessions; attempted feet: 7 out of 14 sessions; navigation: 4 out of 12 sessions). The passive feet movements, evaluated in one patient, yielded mixed results: whereas time-frequency analysis revealed task-related EEG changes over neurophysiological plausible cortical areas, the classification results were not significant enough (*p* < 0.05) to setup an initial classifier for the detection of attempted movements. Concluding, the results presented in this study are consistent with the current state of the art in similar studies, to which we contributed by comparing different types of mental tasks, notably complex motor imagery and attempted feet movements, within patients. Furthermore, we explored new venues, such as an evaluation of passive feet movement as a mean of an initial classifier setup, and rapid delivery of biased feedback.

## Introduction

Functional magnetic resonance imaging (fMRI) studies by Owen et al. (2006) and others Boly et al. (2007), Monti et al. (2010), demonstrating detection of awareness in the unresponsive wakefulness syndrome (UWS, Laureys et al., 2010), paved the way for the development of brain–computer interfaces (BCI) as a means of communication in this patient group. In these studies, patients were asked to imagine playing tennis, or to navigate through their own apartment. Such imaginations led to very specific activations which could then be used to establish a communication channel with people in the minimally conscious state (MCS, Giacino et al., 2002) by means of simple yes/no questions (Monti et al., 2010).

Recent efforts focused on translating these fMRI paradigms to electroencephalography (EEG) technique, as it is widely available, cost effective, and applicable at bedside, even in persons with metal implants. For example, Goldfine et al. (2011) instructed the participants to imagine complex motor and familiar spatial navigation tasks, and analyzed EEG power spectra over a wide range of channels and frequencies. By analysing the EEG power spectra, evidence for performance of mental imagery tasks was found in healthy controls and patients with severe brain injury. In another study, Cruse et al. (2011) asked the participants to imagine movements of their right-hand and toes to command, and analyzed the EEG responses to specific commands. Three of 16 patients (19%) generated repeatedly and reliably suitable EEG responses to two distinct commands, even though they were behaviorally unresponsive. In a follow-up study, addressing some of the methodological challenges, EEG evidence for attempted movements to command was found in an UWS patient (Cruse et al., 2012).

Notable in these efforts are the different approaches to motor tasks—attempted hand/feet movements in Cruse et al. (2012), and complex motor imagery in Goldfine et al. (2011). It is unclear which approach is more suitable, as both have their merits. On one hand, attempted movements lead to well investigated frequency band-specific oscillatory changes over appropriate areas of the sensorimotor cortex (see Pfurtscheller and Da Silva, 1999). On the other hand, imagery of complex movements has been shown to elicit stronger activation than imagery of simple ones with fMRI (Kuhtz-Buschbeck et al., 2003; Boly et al., 2007), encouraging its study with EEG. Furthermore, a recent EEG study performed by Gibson et al. (2014) found that complex and familiar mental tasks can enhance single-trial detectability of imagined movements.

One common challenge facing these EEG efforts is the initial classifier setup for detection of the brain responses. While the delay and variability in brain responses can be addressed with different methods, there is no way of telling whether and when the MCS individuals performed the tasks. However, one could address this challenge by exploiting similarities of the brain responses during passive and attempted movements. In a recent work our group exploited similarities of the sensorimotor EEG changes of the motor cortex during active, passive and imagined movements to setup an initial classifier for the detection of motor imagery in healthy participants (Müller-Putz et al., 2010, 2013a). However, it is an open research question whether this approach is feasible for detection of attempted movements in MCS individuals.

While the current efforts could in theory establish a two-way communication with some of the patients, a real-time feedback on classification of mental imagery with EEG is yet to be evaluated in MCS patients. Such an evaluation is important, as feedback might benefit patient's performance. For example, it is unclear whether rapid delivery of biased (i.e., positive) feedback would benefit patients performing close to chance level, as it has benefited healthy participants (Barbero and Grosse-Wentrup, 2010). Addressing the above mentioned gaps in knowledge—first, an evaluation of both simple and complex motor imagery within patients; second, an evaluation of passive feet movement as a mean of an initial classifier setup; and third, rapid delivery of biased feedback—could provide valuable insights for further development of an EEG based communication device. To that end, the goal of the current work was to investigate whether complex mental imagery, passive, and attempted feet movement can be reliably detected in patients with disorders of consciousness (DoC).

## Materials and methods

### Patients

Six patients diagnosed with MCS took part in this study (one women, five men; age range 21–66 years, mean and standard deviation 41.7 ± 17.8 years). The patients, not in intensive care and in an overall stable medical condition, were selected by the medical staff of the Albert Schweitzer Clinic (Graz, Austria) where all measurements were conducted. Exclusion criteria were gravidity, infections, or participation in other studies. The patients participated in two parts (command following part and online feedback part) with a different number of sessions. The idea was that each patient, if possible, would participate in two session on different days to compensate for possible fluctuations in responsiveness. For patients who participated in more than one session, the follow-up sessions were carried out between 1 and 2 weeks later when possible.

The patients were behaviorally assessed using the Coma Recovery Scale-Revised (CRS-r) within 24 h before or after each EEG measurement in order to keep track of their fluctuations in responsiveness. The CRS-r is composed of 23 items divided into 6 subscales dealing with auditory, visual, motor, oromotor, communication, and arousal functions (Giacino et al., 2004). The standardized scoring has been shown to produce “… reasonably stable scores over repeated assessments…” (Giacino et al., 2009) and is capable of discriminating patients in MCS from those with UWS.

Table 1 provides background and disease related data, as well as the highest estimated CRS-r subscores, of all patients.

**Table 1:** **Overview about participants for both the command following and the online feedback paradigm**.

**Participant**	**Age**	**Sex**	**Onset**
**P1**	**45**	**M**	**April 2010**
Etiology	Traumatic brain injury with craniotomy and evacuation of a traumatic right sighted subdural hematoma, plus a left-sided temporo-parietal subarachnoid hemorrhage, bilateral temporopolar and right-sided temporo-occipital contusion hemorrhages		
Auditory function	Reproducible movement to command		
Visual function	Object recognition		
Motor function	Automatic motor response		
Verbal function	Vocalization/Oral movement		
Communication	Non-functional: intentional		
Arousal	Eye opening w/o stimulation		
Additional diagnoses	Epilepsy, spastic tetraparesis (left more than right), anarthria		
**P2**	**66**	**M**	**March 2011**
Etiology	Traumatic brain injury with left sighted subdural hematoma and left sighted epidural hematoma		
Auditory function	Consistent movement to command		
Visual function	Object localization: reaching		
Motor function	Object manipulation		
Verbal function	Vocalization/Oral movement		
Communication	Non-functional: intentional		
Arousal	Attention		
Additional diagnoses	Epilepsy, tetraparesis (right more than left), dysphagia, anarthria		
**P3**	**21**	**M**	**December 2008**
Etiology	Hypoxic brain injury after resuscitation after mixed drug intoxication		
Auditory function	Reproducible movement to command		
Visual function	Object localization: reaching		
Motor function	Localization to noxious stimulation		
Verbal function	Oral reflexive movement		
Communication	Non-functional: intentional		
Arousal	Eye opening w/o stimulation		
Additional diagnoses	Anarthria, severe spastic tetraparesis		
**P4**	**27**	**M**	**December 2007**
Etiology	Traumatic brain injury with left sighted subdural hematoma and right sighted epidural hematoma, hydrocephalus with ventriculo-peritoneal shunt, st. p. craniectomy left with reimplantation of an artificial bone		
Auditory function	Localization to sound		
Visual function	Visual pursuit		
Motor function	Flexion withdrawal		
Verbal function	Oral reflexive movement		
Communication	None		
Arousal	Attention		
Additional diagnoses	Epilepsy, severe spastic tetraparesis, anarthria		
**P5**	**58**	**F**	**March 2002**
Etiology	Hypoxic brain injury		
Auditory function	Localization to sound		
Visual function	Visual pursuit		
Motor function	Localization to noxious stimulation		
Verbal function	Oral reflexive movement		
Communication	None		
Arousal	Eye opening w/o stimulation		
Additional diagnoses	Spastic tetraparesis, osteoporosis		
**P6**	**33**	**M**	**January 2002**
Etiology	Traumatic brain injury after car accident		
Auditory function	Localization to sound		
Visual function	Visual pursuit		
Motor function	Flexion withdrawal		
Verbal function	Oral reflexive movement		
Communication	Non-functional: intentional		
Arousal	Eye opening w/o stimulation		
Additional diagnoses			

Informed consent was obtained from the patient's legal representatives. The study was approved by the local ethics committee (Medical University of Graz) and is in accordance with the ethical standards of the Declaration of Helsinki.

### Experimental paradigms

The study consisted of two parts. The first part (performed by 4 patients; age range 21–66, mean (μ) and standard deviation (σ) 39.8 ± 20.3 years, all men) comprised a command following paradigm. The second part was an online paradigm which was performed by 4 patients (one women and 3 men; age range 27–66 years, μ and σ 46.0 ± 18.9 years), of which two already participated in the first part.

#### Command following paradigm

**Figure 1:**
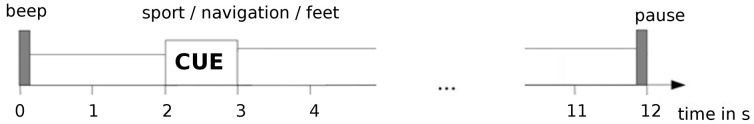
**Experimental paradigm for measurements in patients**. Timeline of a single trial is shown here.

Within an experimental session, up to four different tasks (i.e., sport, navigation, attempted/passive feet movement) were performed in a block design. Each task was performed during three consecutive runs, with each run having 15 cue-based trials (auditory cue) of 12 s length, yielding 45 trials/task (see Figure 1). At the beginning of a trial a beep tone was given. After 2 s, an auditory cue, generated by a text-to-speech synthesizer, was delivered via in-ear headphones. The cue was a verbal instruction to perform the current task (i.e., either “sport,” “navigation,” or “feet”) lasting for 1 s. For the “passive feet” task, no cue was given to the patients, as it was only audible to the caregiver performing the passive feet movement. Between the trials a random pause (also auditorily indicated) of 4–6 s length was given. Detailed verbal instructions were given to the participant by the experimenter before the measurement started. The purpose of these instructions, repeated before each run, was to inform the patient about the tasks he/she has to perform. The order of the tasks was pseudo randomized across the measurement sessions. Each measurement session was conducted on a separate day.

In more detail, for the “sport” task the participants were instructed to imagine performing one sport of their choice in the first person perspective. For measurements with non-responsive patients there is no way of knowing for sure which sport they chose. However, they were instructed to keep their choice while performing this task. For the “navigation” task the participants were instructed to imagine navigating through their house, looking around each room, without focusing on the movement. For the “feet” task the participants were instructed to repeatedly attempt feet dorsiflexion (i.e., several consecutive attempts during a single trial). In the “passive feet” task, a caregiver performed a brisk (i.e., ~1 s long) dorsiflexion of both feet. The cue was the same as for the “feet” task, but it was only audible to the caregiver.

#### Online feedback paradigm

The online feedback paradigm built upon the command following paradigm by introducing feedback. In general the transition from offline to online paradigm within a measurement session was possible but contingent upon results (i.e., accuracy, confusion matrix), statistical significance (i.e., number of trials, leave-one-out or blockwise crossvalidation), plausibility of results (i.e., neurophysiological plausible EEG channels), and patient's condition (i.e., fatigue, indicated by an obviously reduced vigilance). To that end, it started with recording of a few minutes resting state EEG, followed by a run of command following paradigm without feedback, and afterwards an initial classifier setup. The next step was contingent upon the estimated accuracy and patient's condition. In case of promising results, the next run was for the online feedback paradigm, again followed by a classifier setup in order to obtain a more reliable estimate of the accuracy. This step (i.e., a run of online feedback paradigm, followed by a classifier setup) was repeated depending on the estimated accuracy and patient's condition. Furthermore, the following changes were made compared to the initial command following paradigm: (i) only motor tasks (i.e., sport, attempted feet) were employed, based on offline analysis of shared common patient data recorded in the command following paradigm (Müller-Putz et al., 2013b); (ii) a varying number of trials, separated in blocks of 15 trials by short breaks, were recorded for each task; (iii) in case the initial command following led to online feedback, the second task was discarded.

### Recording

**Figure 2:**
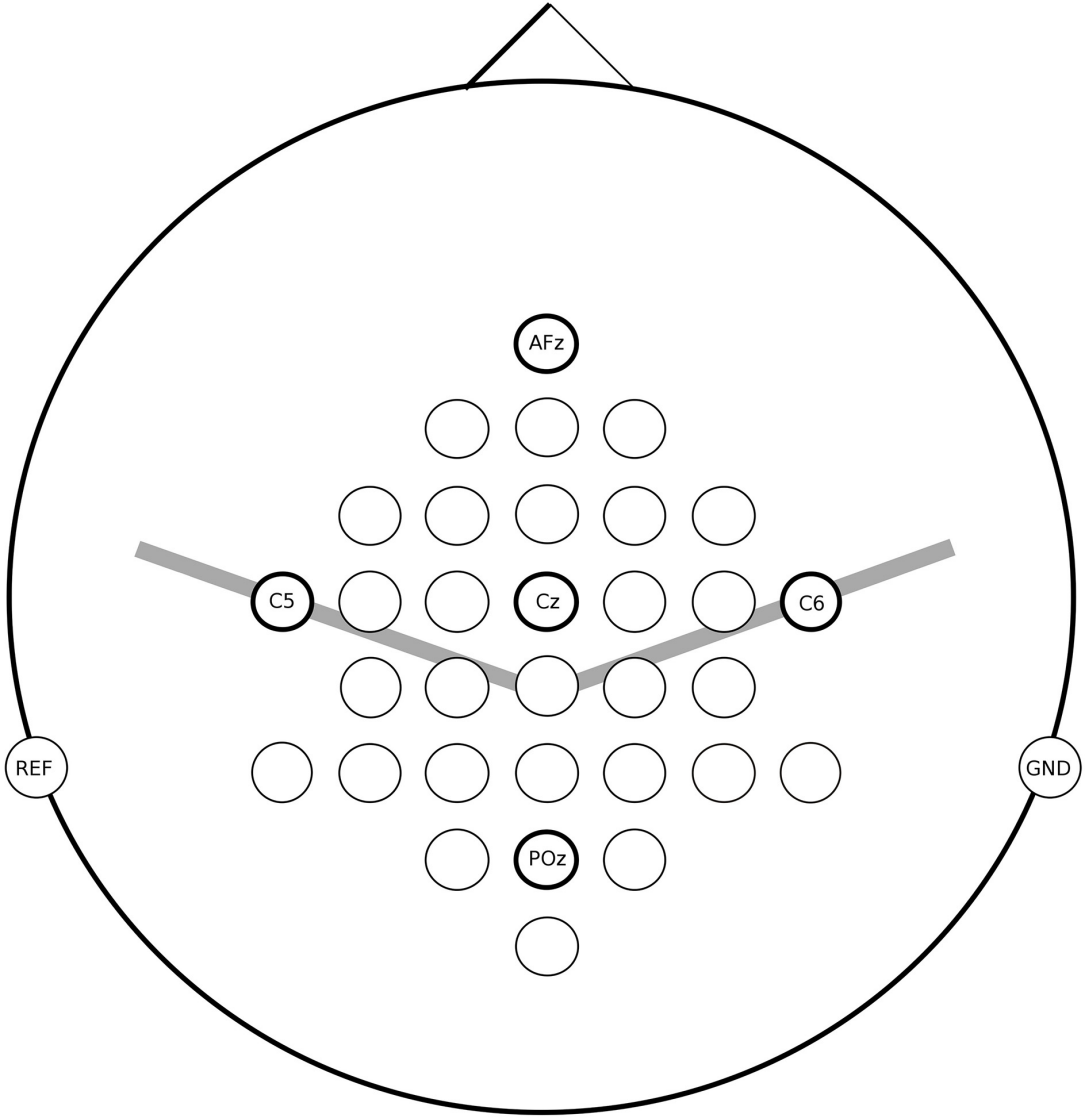
**EEG channel locations used for measurements in patients**.

For all measurements the EEG was recorded from 32 active electrodes (g.tec, Guger Technologies, Austria) located over frontal, central and parietal areas (for details see Figure 2). The signals were acquired with a g.UBSamp amplifier (Guger Technologies, Austria) with 512 Hz sampling rate, 0.5 Hz high-pass, and 100 Hz low-pass filter, and an additional 50 Hz notch filter.

### Data analysis

#### Preprocessing

For the offline analysis, artifacts were removed from EEG with an elaborate projection method which automatically detects neuronal and artifactual source components derived from independent component analysis (ICA). We used the binary Infomax independent component analysis by Enghoff (1999), based on the Matlab version of Scott Makeig and collaborators, to separate EEG and EOG signals into independent components (Makeig et al., 1996). We identified independent components (ICs) representing eye movements, eye blinks, and muscle activity by visual inspection using methods described in McMenamin et al. (2010), and removed them. We multiplied the remaining components by the mixing matrix produced by the ICA algorithm to reconstruct cleaned EEG.

For the online feedback delivery, due to time and resources constraints (i.e., short breaks between consecutive runs and a single laptop certified for clinical measurements, respectively) artifacts were rejected. To that end, muscle and movement artifacts, as well as other transient non-stationarities in the ongoing EEG signals, were detected by inverse filtering of orthogonal Laplacian derivation (Scherer, 2008). Autoregressive (AR) parameters of the inverse filter were estimated from a 1 to 2 min segment of resting state EEG, recorded at the beginning of each session. The detection threshold was defined as five times Root-Mean-Square from the resting-state EEG. Trials in which the detection threshold was exceeded were discarded from the analysis.

#### Time-frequency analysis (ERD/ERS calculation)

Event-related desynchronization (ERD) and event-related synchronization (ERS) are defined as the percentage of power decrease (ERD) or power increase (ERS) in a defined frequency band in relation to a reference interval (Pfurtscheller and Da Silva, 1999). To analyze the percentage of power decrease (ERD) or power increase (ERS) relative to a reference interval (second 1–2 in the paradigm), time-frequency map for frequency bands between 6 and 40 Hz (35 overlapping bands using a band width of 2 Hz with a step size of 1 Hz) was calculated (Graimann, 2002). Logarithmic band power features, calculated by band-pass filtering, squaring and subsequently averaging over the trials, were used to assess changes in the frequency domain. To determine the statistical significance of the ERD/ERS values a t-percentile bootstrap algorithm with a significance level of α = 0.05 was applied (Davison and Hinkley, 1997). In the ERD/ERS maps statistically significant ERD values were plotted as red dots and significant ERS values as blue dots.

#### Feature extraction and classification

***Feature extraction***. Logarithmic band power features were calculated for multiple frequency bands (θ: 4–7 Hz; α: 7–13 Hz; β_L_: 13–19 Hz; β_M_: 19–25 Hz; β_H_: 25–30 Hz) by band-pass filtering, squaring and averaging over 1 s in a sample by sample way.

For further analysis, a trial was divided into consecutive, non-overlapping time periods of 1 s duration. One time period, from *t* = 1 s to *t* = 2 s (i.e., 1 s before the cue onset), was designated as the reference. Finally, a single value was sampled at the middle of each time period, and was used in the subsequent classification.

***Classification***. We sought to identify one Laplacian channel/frequency band yielding the best results for the current task. Thus, we estimated the accuracy over different time periods relative to the reference, for each of the frequency bands (i.e., θ, α, β_L_, β_M_, β_H_), and at each of the Laplacian channels. To that end we used a linear discriminant analysis (LDA) classifier.

To avoid overfitting cross-validation was applied to estimate the accuracy. For the offline analysis, a nested block-wise cross-validation (10 × 10 inner fold; leave-one-out-block outer fold) was applied. For the online paradigm, both leave-one-trial-out (initial runs), as well as nested blockwise (10 × 10 inner fold; leave-one-out-block outer fold; micro-averaging of confusion matrices) cross-validation were applied. Also, the classifier was recalculated following each run, based on the EEG recording from up to three previous runs.

To ensure comparable results, we performed a separate cross-validation for each channel using comparable data (i.e., randomized trial indices in inner/outer folds were held constant). Furthermore, in each cross-validation, classification was performed separately for each frequency band and time segment.

#### Online feedback

Feedback was only given for correct classified trials. The feedback was either “Sport/feet correctly recognized” in the case of correct classifier prediction for more than 50% of the duration of the imagery period in the trial (Daly et al., 2013a), or “Pause” otherwise (also for the trials in which EEG artifacts were detected).

## Results

Tables 2, 3 show *post-hoc* analysis results of the command following paradigm and online paradigm, respectively. The Laplacian channel derivation and the frequency band yielding the highest accuracy, as estimated with the blockwise nested crossvalidation, is reported. The reported results were obtained with respect to a baseline reference period, and no differentiation between the tasks was made.

**Table 2:** **Summary of results for the offline detection of different tasks for the command following paradigm**.

**Participant/Session no**.	**CRS-r score**	**Sport**	**Navigation**	**Attempted feet**
P1/1	18	71% (CP1, ϑ, 0.01)	n.s.	73% (C2, α, 0.01)
2	18	n.s.	n.s.	n.s.
3	17	n.p.	n.s.	n.p.
4	19	65% (CPz, ϑ, 0.05)	n.s.	n.s.
P2/1	14	76% (Fz, ϑ, 0.01)	n.s.	69% (FC1, α, 0.01)
2	15	n.s.	72% (P3, ϑ, 0.01)	n.s.
3	14	65% (C2, ϑ, 0.05)	n.p.	80% (FC1, ϑ, 0.01)
P3/1	14	n.s.	n.s.	n.s.
2	13	66% (CP1, ϑ, 0.05)	n.s.	65% (CP1, β_M_, 0.05)
3	13	n.s.	72% (POz, β_M_, 0.01)	68% (Cz, ϑ, 0.05)
P4/1	9	66% (Fz, α, 0.05)	n.s.	n.p.
2	11	n.s.	72% (C4, β_M_, 0.01)	n.s.
3	11	n.s.	72% (C2, β_M_, 0.01)	64% (Fz, β_M_, 0.05)

*Discrimination between mental imagery task/attempted feet/passive feet movement, and the reference (1 s before the cue onset). Only significant (p = 0.01 and/or p = 0.05, considering the number of trials, Müller-Putz et al., 2008) accuracy is reported. CRS-r, Coma Recovery Scale-Revised; acc (ch, band, p), accuracy (Laplacian channel, band, significance level); n.s., not significant; n.p., not performed*.

**Table 3:** **Summary of results for the *post-hoc* offline detection of different tasks for the online feedback paradigm**.

**Participant/Session no**.	**CRS-r score**	**Sport**	**Attempted feet**
P2/1	18	n.s.	n.p.
2	17	68% (CP2, α, 0.01)	n.p.
P4/1	11	64% (Fz, ϑ, 0.05)	n.p.
2	11	65% (FC2, β_M_, 0.05)	n.p.
P5/1	11	n.p.	n.s.
2	11	n.p.	n.s.
P6/1	11	n.s.	64% (CP2, β_M_, 0.05)
2	12	71% (C3, β_M_, 0.01)	n.p.

*Discrimination between motor imagery task/attempted feet movement, and the reference (1 s before the cue onset). Only significant (p = 0.01 and/or p = 0.05, considering the number of trials, Müller-Putz et al., 2008) accuracy is reported. CRS-r, Coma Recovery Scale-Revised; acc (ch, band, p), accuracy (Laplacian channel, band, significance level); n.s., not significant; n.p., not performed*.

In both the command following and the online feedback paradigm, offline classification accuracies above chance were reached in all three tasks (i.e., attempted feet, sport, and navigation), with motor tasks yielding significant results more often than navigation (sport: 10 out of 18 sessions; attempted feet: 7 out of 14 sessions; navigation: 4 out of 12 sessions). In the online feedback paradigm, *post-hoc* classification accuracies above chance (*p* = 5%) were reached by three out of four patients in either the attempted feet (F) or sport (S) task. Online accuracies, as used for the feedback delivery, were below the level of significance (i.e., random) and are not reported.

**Figure 3:**
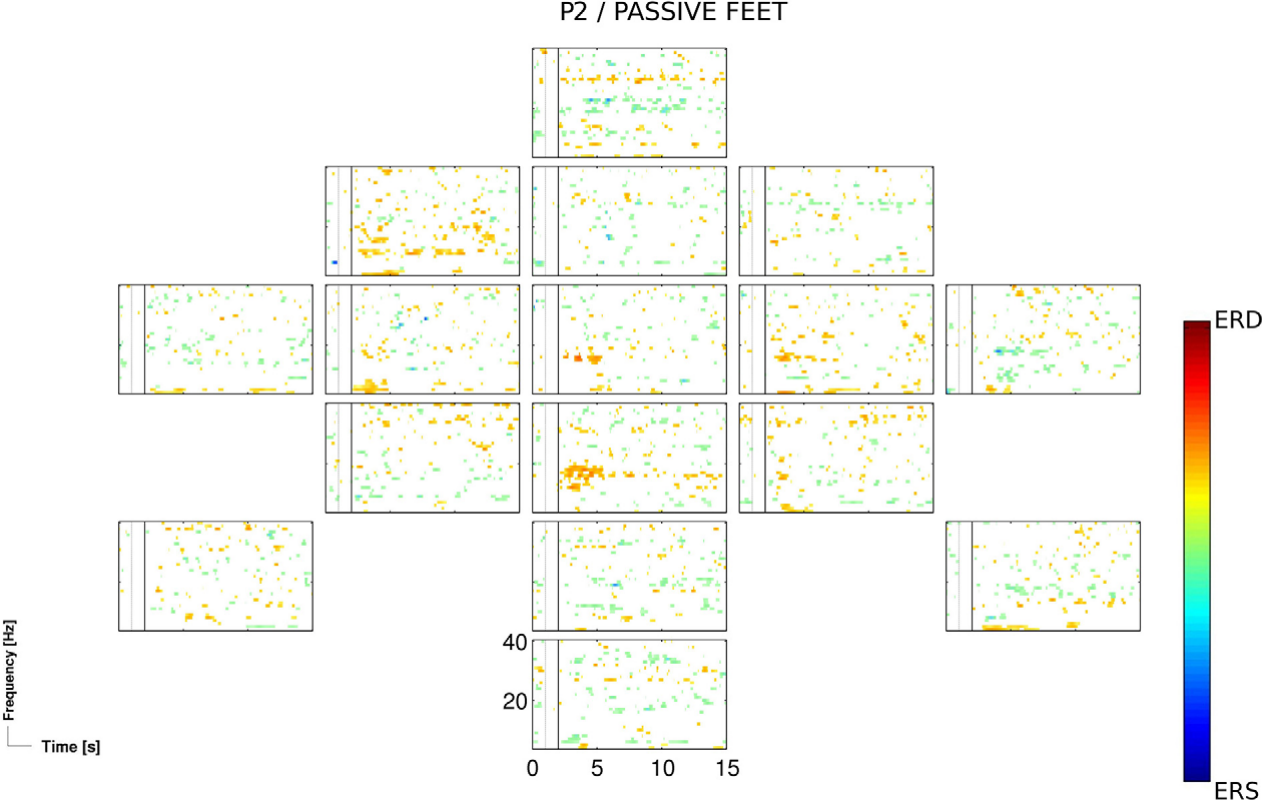
**ERD/S map for the participant P2 and for the passive feet condition of the 3rd session, calculated for Laplacian channel derivations**.

The passive feet movements, evaluated once in the third session of patients P2, did not yield significant accuracies. However, time-frequency analysis revealed task-related EEG changes over neurophysiological plausible cortical areas (Figure 3).

## Discussion

In the current work involving patients with DoC our aim was threefold: (i) to evaluate different types of motor imagery; (ii) to evaluate passive feet movements as a mean of an initial classifier setup; and (iii) to evaluate rapid delivery of biased feedback. To that end, we investigated whether complex mental imagery, attempted, and passive feet movements can be reliably detected in patients with disorders of consciousness (DoC).

**Figure 4:**
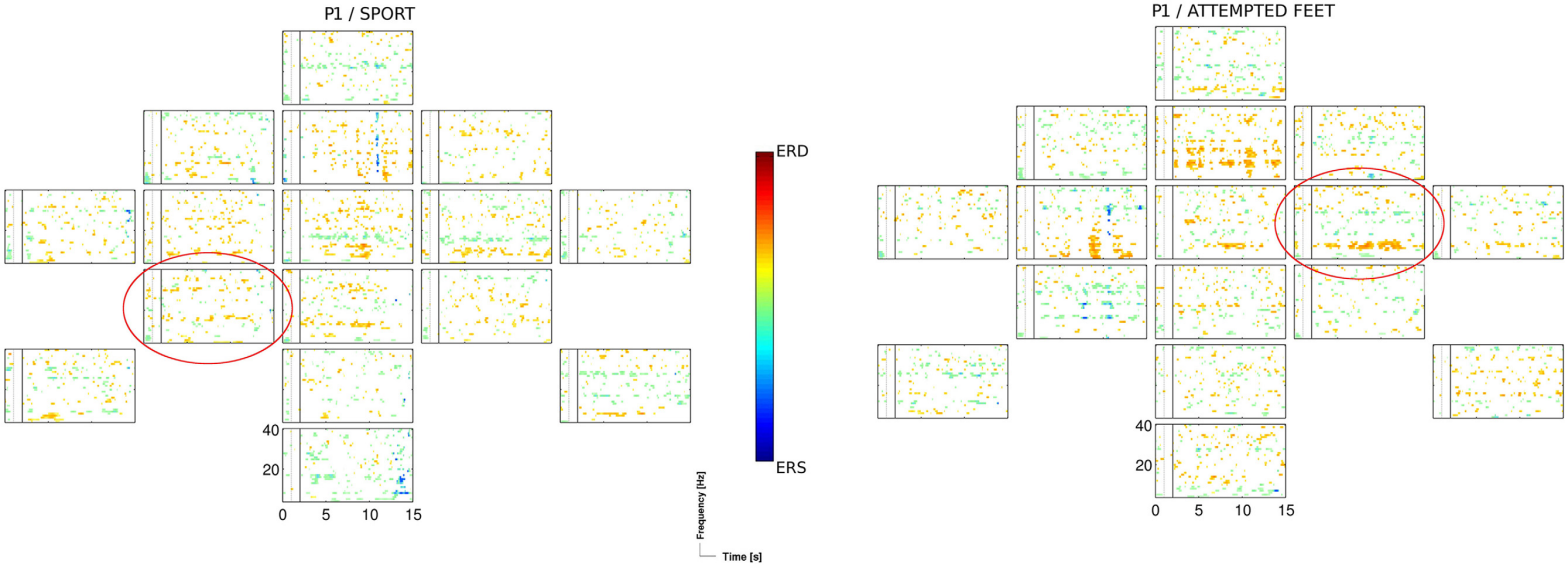
**ERD/S map for the participant P1 and for the sport, and the attempted feet task of the 1st session, calculated for Laplacian channel derivations**. Marked with the red circle is the Laplacian channel derivation yielding the highest accuracy, as estimated with the blockwise nested crossvalidation.

The two motor tasks, the sport imagery and attempted feet movement, accounted for almost two thirds (i.e., 62%) of sessions yielding significant (*p* < 0.05) accuracies, with similar outcomes within sessions. This is in line with previous findings indicating that, among other tasks, motor imagery rather than spatial navigation most frequently results in better classification performance (Friedrich et al., 2012). The sport imagery resulted in activations in theta (centro-parietal, central, and frontal), alpha (centro-parietal, frontal), and middle beta band (fronto-central, central). The attempted feet resulted in activations in theta (fronto-central, central), alpha (central), and middle beta band (centro-parietal, frontal). In Figure 4, ERDS patterns for the sport and attempted feet tasks are exemplified for the participant P1 and the first session.

The passive feet movements were evaluated in only one out of four patients (P2), as an evaluation in other patients was not feasible due to their medical conditions (i.e., spasticity). The evaluation in P2 yielded mixed results: on one hand, time-frequency analysis revealed task-related EEG changes over neurophysiological plausible cortical areas (Figure 3); on the other hand, classification results were not significant enough (*p* < 0.05) to setup an initial classifier for the detection of attempted movements. However, the attempted feet movements performed after the passive feet movements yielded highly significant (*p* < 0.01) accuracies, prompting the question whether this was more than a mere coincidence.

The online feedback paradigm led to ERDS patterns in MCS patients that, when analyzed *post-hoc*, could be detected at around 70% accuracy with blockwise crossvalidation. However, online detection of these ERDS patterns was at the random level only. One possible explanation for this discrepancy is that, while a longer mental imagery period may be beneficial for inducing the desired ERDS patterns, a shorter detection period may be needed in order to reliably detect these patterns. In the latter case, a continuous auditory feedback may be more suitable than a discrete auditory feedback. Further investigation is needed to assess whether and to what extent the MCS patients could benefit from an auditory feedback.

In Cruse et al. (2011) consistent and robust responses to command for attempted movements were observed in the EEG of 5 out of 23 of the MCS patients. Similarly, we estimated highly significant (i.e., *p* = 0.01) accuracy for attempted feet movements in two out of six of the MCS patients. Worth pointing out is that we employed longer trials to accommodate for more complex mental imagery tasks. In Goldfine et al. (2011) two out of three patients (one patient in MCS and one in LIS) showed evidence of motor imagery task performance, which is similar to our findings with 62% (*N* = 21) of sessions yielding significant (*p* < 0.05) accuracies for either sport or attempted feet task.

**Figure 5:**
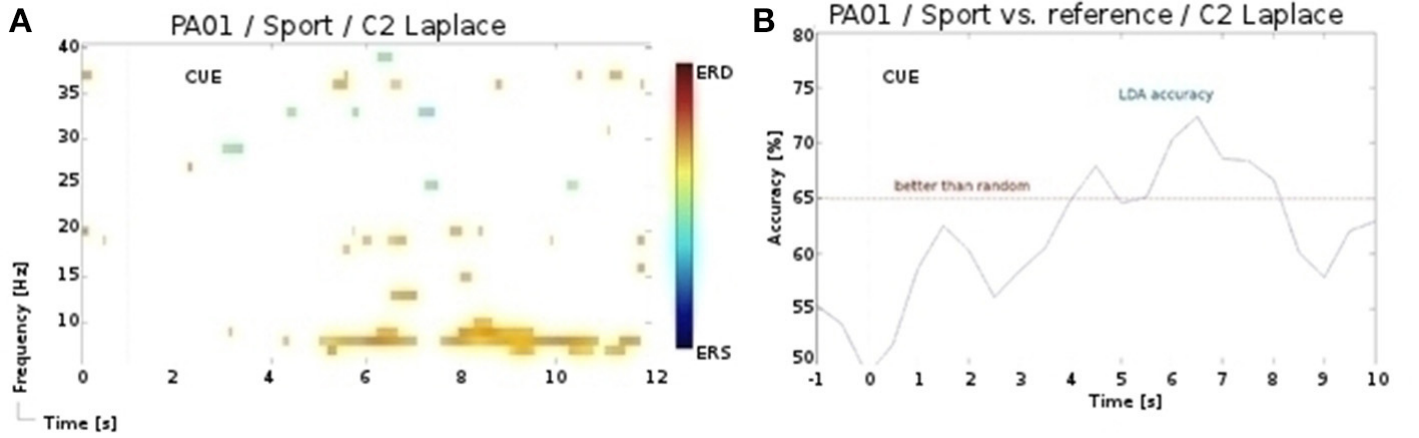
**(A)** ERD/S map for the participant P1 and for the sport task of the 2nd session. **(B)** LDA accuracy over trial duration for this participant, sport task, and α band during the 2nd session.

In our initial analysis (Müller-Putz et al., 2013b), we employed manual artifact rejection instead of the ICA, and obtained partially different results. Notably, for the participant P1 and for the sport task of the second session we found activation over central sensorimotor area (see Figure 5), yielding significant (*p* < 0.01) accuracies. However, following the ICA artifact rejection, the significance of these patterns diminished. In only one additional, case namely for the participant P6 and for the sport task of the first session, did we observe a similar discrepancy in results. One explanation for these discrepancies is, that the rejected electromyography (EMG) components also entailed the signal of interest, i.e., discriminative periods of neural activity (McMenamin et al., 2010). In contrast, for the navigation task significant accuracies were obtained only after the ICA preprocessing, as this task was especially prone to artifacts. Whereas in healthy participants these issues can be addressed by rejecting the artifactual EEG, doing so in the patients is rarely an option, as it is often ridden with artifacts. Therefore, we are aiming to address these issues with an automated and online artifact removal method, combining wavelet decomposition, independent component analysis, and thresholding (Daly et al., 2012, 2013b, 2014).

The above mentioned tasks were chosen due to previous investigations: for example the passive and attempted movement conditions were already investigated by our group in studies with healthy subjects (Müller-Putz et al., 2007, 2013a; Solis Escalante et al., 2012). In Müller-Putz et al. (2013a) 10 healthy subjects performed brisk passive feet/hand movements and reached mean offline classification accuracies of 81% (±14) and 76% (±13) for passive hand and feet task, respectively. In Müller-Putz et al. (2007) EEG-changes during passive and attempted foot movements were investigated in 10 healthy subjects and seven patients suffering from a complete sensor and motor paralysis. In this study healthy subjects showed distinctive ERD/ERS patterns similar to earlier studies focusing on active movements (Neuper and Pfurtscheller, 1996, 2001; Stancak et al., 2000; Müller et al., 2003) and passive movements (Cassim et al., 2001; Müller et al., 2003). Furthermore, in in five out of seven patients during attempted movement diffuse ERD/ERS patterns were found. Finally, attempted movements were already used by Cruse et al. (2012) to detect awareness in a patient who had been diagnosed to be in UWS.

In the command following paradigm we opted for a block-design instead of a pseudo randomized design mainly for the following two reasons: first, we wanted to reduce the cognitive demand by performing only one condition at a time, instead of pseudo randomizing up to four different conditions (i.e., sport, attempted feet, navigation, and passive feet); second, in case a measurement session had to be ended prematurely (e.g., due to patients obvious reduced vigilance) block design would increase the probability that at least for some of the conditions (i.e., the initial ones) enough data has been gathered. We reduced the risk of the task-irrelevant intrablock correlations in the EEG significantly accounting for the classification results through: (i) rigorous removal of artifacts with ICA; (ii) use of a simple and robust classifier with few features; (iii) control for physiological plausibility of results by means of time-frequency analysis.

It is important to note, that even though the results presented in this study are consistent with the current state of the art in similar studies (Cruse et al., 2011; Goldfine et al., 2011), a functional and accurate communication with MCS patients, as demonstrated with fMRI, is yet to be achieved with EEG and will be the primary goal of our further investigations.

Concluding, we contributed to the state of the art by comparing different types of mental tasks, notably complex motor imagery and attempted feet movements, within patients. Furthermore, we explored new venues, such as an evaluation of passive feet movement as a mean of an initial classifier setup, and rapid delivery of biased feedback. Further application of online feedback, as well as of an auditory scanning method, as described recently in Müller-Putz et al. (2013a), has to be investigated.

### Conflict of interest statement

The authors declare that the research was conducted in the absence of any commercial or financial relationships that could be construed as a potential conflict of interest.
