# Supplementary figures and images for: Detection of mental imagery and attempted movements in patients with disorders of consciousness using EEG
Source: Front Hum Neurosci. 2014 Dec 12;8:1009. doi: 10.3389/fnhum.2014.01009 (PMC4264500; doi:10.3389/fnhum.2014.01009)

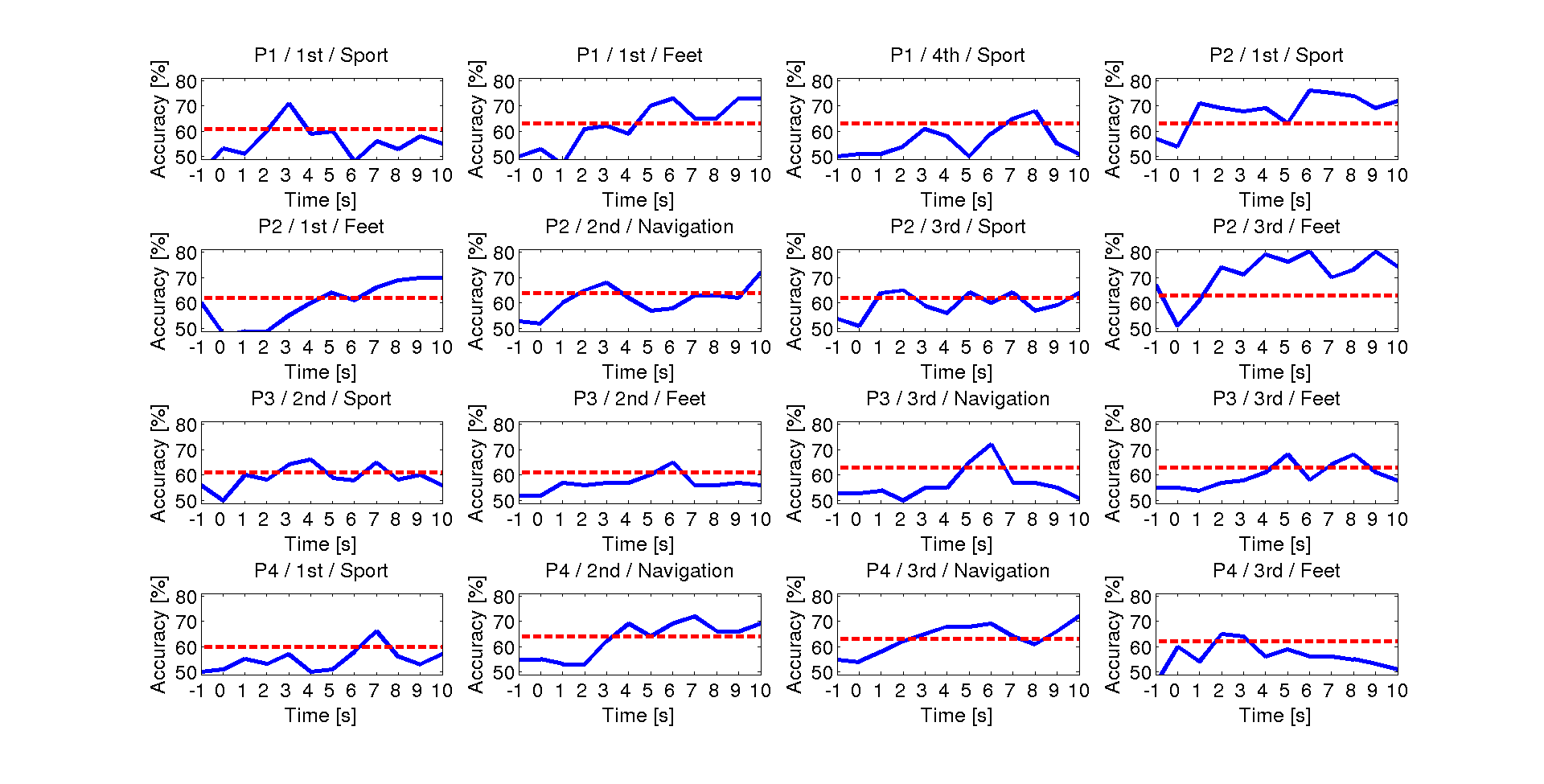

Supplement: Supplementary file 1 [file Image1.PNG]

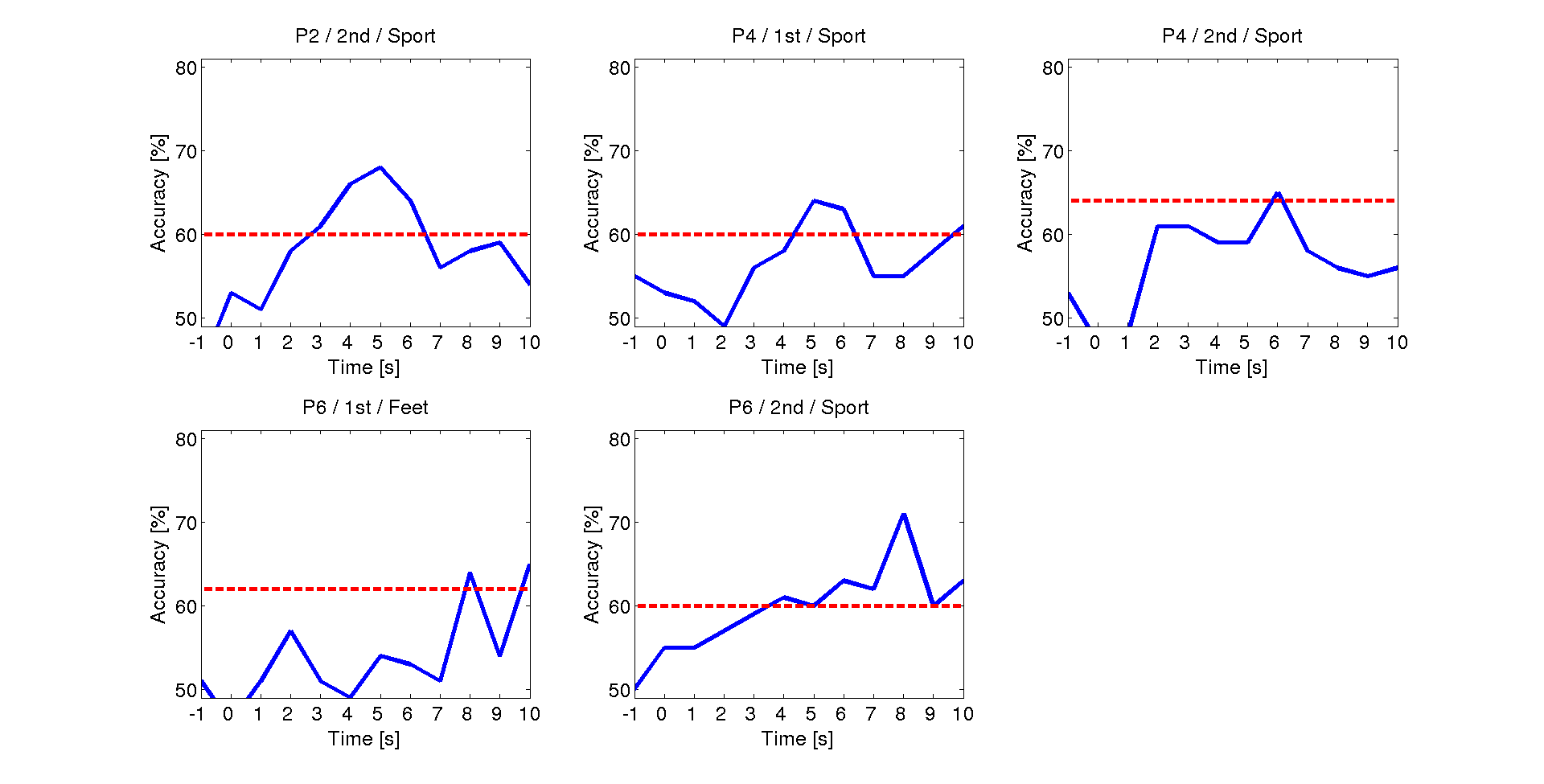

Supplement: Supplementary file 2 [file Image2.PNG]

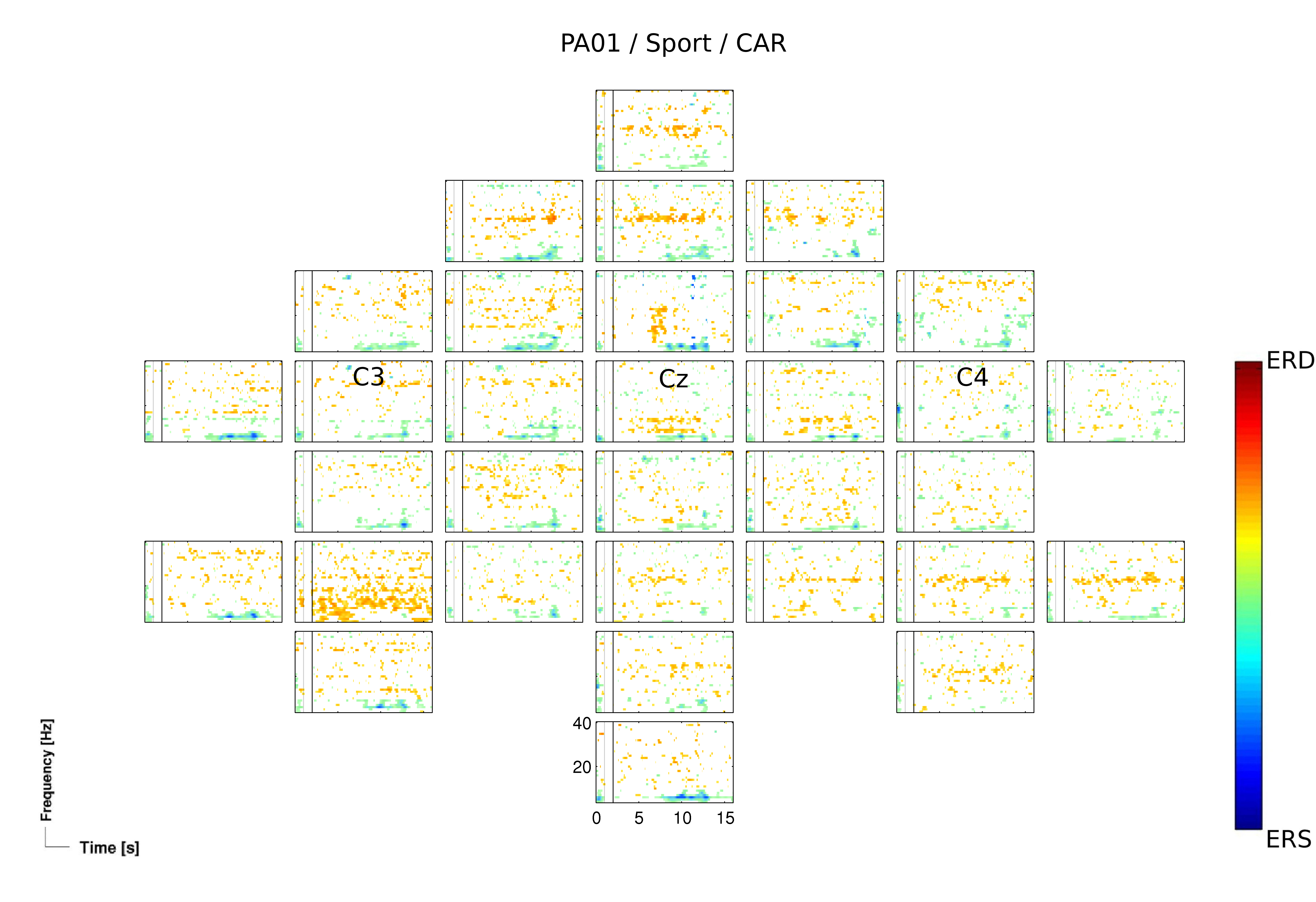

Supplement: Supplementary file 3 [file Image3.PNG]
